# Telehealth Utilization in High-Risk Pregnancies During COVID-19

**DOI:** 10.1089/tmr.2023.0006

**Published:** 2023-05-15

**Authors:** Margie A. Rayford, Joshua M. Morris, Ramona Phinehas, Elizabeth Schneider, Amanda Lund, Sarah Baxley, Jim Y. Wan, Patricia J. Goedecke, Roberto Levi-D'Ancona

**Affiliations:** ^1^ Department of Obstetrics and Gynecology, and University of Tennessee Health Science Center, Memphis, Tennessee, USA.; ^2^ Department of Preventive Medicine, University of Tennessee Health Science Center, Memphis, Tennessee, USA.; ^3^ Department of Obstetrics and Gynecology, Southern Illinois University, Springfield, Illinois, USA.

**Keywords:** telehealth, telemedicine, obstetrics, high risk

## Abstract

**Purpose:**

To determine how telehealth has influenced outcomes in high-risk obstetrics patients during the Coronavirus disease 2019 (COVID-19) pandemic.

**Methods:**

A retrospective chart review was conducted to identify patterns in both telehealth and in-person clinic visits among patients of a Maternal Fetal Medicine (MFM) department from the onset of the COVID-19 pandemic from March 2020 until October 2021. For the descriptive analysis, *p*-values were calculated using Wilcoxon rank sum for continuous variables and chi-square or Fisher exact (where cell *n* < 5) for categorical variables. Variables of interest were then tested for their univariate association with telehealth utilization using logistic regression. Variables found to meet the criterion of *p* < 0.2 in the univariate case were introduced into a multivariable logistic model with a backward elimination for determining variable retention. We aimed to analyze whether telehealth visits significantly impacted pregnancy outcomes.

**Results:**

Four hundred nineteen high-risk patients visited the clinic via in-person and/or telehealth appointments during the study period: 320 patients without telehealth visits and 99 patients with telehealth visits. Care provided by telehealth visits was not found to be related to self-reported race (*p* = 0.81), maternal body mass index (*p* = 1.0), or maternal age (*p* = 0.53). Patients with private insurance were more likely to have telehealth visits than patients with public insurance (79.9% vs. 65.5%, *p* < 0.01). In univariate logistic analyses, patients with diagnoses of anxiety (*p* < 0.01), asthma (*p* = 0.03), and depression (*p* < 0.01), at the time care was established, were more likely to have telehealth visits. Those patients with telehealth visits did not have any statistical differences in mode of delivery (*p* = 0.2) or pregnancy outcomes (*p* = 0.12), including fetal demise, preterm delivery, or delivery at term as compared with patients with all in-office visits. In multivariable analysis, patient conditions of anxiety (*p* < 0.01), maternal obesity (*p* < 0.01), and twin pregnancy (*p* = 0.04) were associated with higher rates of telehealth visits.

**Conclusion:**

Patients with certain pregnancy complications elected to have more telehealth visits. Patients with private insurance were more likely to have telehealth visits than patients with public insurance. There are benefits for patients with certain pregnancy complications to incorporate telehealth visits in addition to regularly scheduled in-person clinic visits and may be suitable in a post-pandemic setting as well. Further research in this field is needed to better understand the impact of implementing telehealth in high-risk obstetrics patients.

## Introduction

Before the Coronavirus disease 2019 (COVID-19) pandemic, telehealth was increasingly promoted as an opportunity to expand health care access to rural and underserved populations.^1–3^ In addition, telehealth was viewed as an intervention to substantially reduce health care costs.^4^ However, due to the COVID-19 pandemic and the need to provide consistent reliable care to patients with the concurrent need to provide care within safe distancing guidelines, telehealth was urgently adopted as a safer means of providing regular and consistent medical care.^5^

One of the Centers for Disease Control and Prevention (CDC) recommendations was the implementation of telehealth to mitigate risks of COVID-19 viral exposure to patients while ensuring continued care in both inpatient and outpatient settings.^6^ The CDC emphasized and encouraged patients with a high risk of severe illness to seek telehealth options when available. The pressure to provide consistent care while not endangering those at a higher risk of viral sequela were acutely felt while caring for pregnant individuals, as pregnancy itself is considered a baseline immunocompromised state.

The COVID-19 pandemic further increased immune vulnerability in this population. A recent meta-analysis found that COVID-19 infection in pregnancy was associated with a higher rate of preterm birth, preeclampsia, cesarean delivery, and perinatal death with preterm birth being the most common outcome.^5^

Telehealth has seen expanded use in prenatal care and has been shown to be both safe and effective.^7–9^ Several studies have revealed that telehealth enhances patient satisfaction,^10–12^ while maintaining comparable maternal and fetal outcomes to in-person visits.^13,14^ A study by Peahl et al. suggested that “low-risk” patients prefer telehealth visits over in-person visits when available.^15^

Further, a recent systemic review found that telehealth interventions were associated with improvements in obstetric outcomes, decreased perinatal smoking, increased breastfeeding, and improved schedule optimization for high-risk obstetrics patients and providers.^16^

Telehealth may also further improve health care access for disadvantaged populations who are at a higher risk of complications due to socioeconomic status and access to health care. However, studies have been inconclusive and have instead suggested that obstetric patients with a lower socioeconomic status and/or public health insurance were less likely to access telehealth for prenatal care.^17^ Further research is, therefore, needed to help clinicians determine how to integrate telehealth into practice in ways that improve patient care in disadvantaged populations.

Following the CDC guidelines, telehealth outpatient visits were implemented in our tertiary academic medical center Maternal Fetal Medicine (MFM or “high-risk”) outpatient clinic. Our study specifically evaluates pregnant individuals referred to be seen by an MFM specialist focusing on managing diagnoses, contributing to the classification of a high-risk pregnancy. The purpose of this study was to review the impact of telehealth for high-risk obstetric patients receiving care at a single tertiary academic center during the COVID-19 pandemic. We hypothesized that care and subsequent outcomes attributed to patients who sought care via telehealth would be noninferior to patients who continued with standard all in-person office visits.

## Methods

A retrospective chart review was conducted to identify all patients who had either telehealth, telehealth and in-person visits, or in-person only visits in an MFM department from the onset of the COVID-19 pandemic through the end of the study period (March 2020 until October 2021). At the onset of the pandemic, clinic visits were limited, and patients were automatically assigned a telehealth visit by providers. As time progressed, patients were given the choice to have either in-office or telehealth visits at the conclusion of their visit. Doximity (Doximity, Inc., San Francisco, CA), a Health Insurance Portability and Accountability Act-compliant audio and visual messaging software, was used to conduct telehealth visits either by combined audio-visual or by audio-only calls according to individual provider preference.

Providers included MFM physicians and nurse practitioners as well as obstetrics and gynecology resident physicians training with MFM physicians. This study received Institutional Review Board approval from the University of Tennessee Health Sciences Center (IRB# 21-08173-XP) before initiation of this study. All individuals aged 13 to 42 years old with a high-risk pregnancy seen at the MFM office during the study period were included.

Data obtained included maternal age at time of first clinic appointment, all medical diagnoses pertaining to pregnancy, self-reported race, type of insurance, number of telehealth visits, body mass index (BMI) (defined as kg/m^2^), mode of delivery, pregnancy outcomes (live birth rate and intrauterine fetal demise), and total incidence of fetal demise. We aimed at analyzing whether telehealth significantly impacted pregnancy outcomes according to patient pregnancy complications and known medical diagnoses.

Pregnancy outcomes and covariates of interest were described by telehealth utilization status using number and row percentage for categorical variables and mean, median, interquartile range, and standard deviation for continuous variables (Table 1). In preliminary comparisons of patients with telehealth and in-clinic only patients, Chi-square or Fisher exact test (where cell *n* < 5) were applied to categorical variables. Wilcoxon rank sum tests were applied to continuous variables.

**Table 1.** Demographics Characteristics

Characteristics	Controls (*N* = 320, %)	Telehealth (*N* = 99, %)	*p*
Race			0.81
Black	232 (72.5)	74 (74.7)	
White	54 (16.9)	17 (17.2)	
Hispanic	26 (8.1)	7 (7.1)	
Other	8 (2.5)	1 (1.0)	
Pregnancy outcome			0.12
Successful delivery	235 (73.4)	80 (80.8)	
Not delivered	32 (10.0)	3 (3.0)	
Fetal demise	3 (0.9)	2 (2.0)	
Unknown	50 (15.6)	14 (14.1)	
Type of insurance			*<0.01*
Blue Cross Blue Shield	106 (33.2)	31 (31.6)	
Cigna	14 (4.4)	7 (7.1)	
United health care	135 (42.3)	26 (26.5)	
Other	64 (20.1)	25 (25.5)	
Unknown	0 (0.0)	9 (9.2)	
Mode of delivery			0.20
Vaginal	106 (33.1)	39 (40.2)	
Cesarean section	130 (40.6)	33 (34.0)	
Outcome unknown	84 (26.2)	27 (27.3)	
Pregnancy complications			
Anemia	34 (10.6)	13 (13.5)	0.43
Chronic hypertension	85 (26.6)	22 (22.9)	0.47
Type I diabetes	9 (2.8)	3 (3.1)	0.87
Type II diabetes	37 (11.6)	12 (12.5)	0.80
Gestational diabetes	51 (15.9)	16 (16.7)	0.86
Asthma	49 (15.3)	24 (25.0)	*0.03*
Depression	14 (4.4)	12 (12.5)	*<0.01*
Anxiety	12 (3.8)	11 (11.5)	*<0.01*
History of deep vein thrombosis	10 (3.1)	3 (3.1)	1.00
Twin pregnancy	6 (1.9)	5 (5.2)	0.07
Preeclampsia w/o SF	11 (3.4)	10 (10.4)	0.74
Preeclampsia w/SF	14 (4.4)	4 (4.2)	*0.03*
Gestational hypertension	12 (3.8)	5 (5.3)	0.51
Thyroid disorder	13 (4.1)	7 (7.3)	0.19
Other	175 (54.7)	45 (46.9)	0.18
Body mass index			1.00
<30	98 (30.7)	30 (30.6)	
30–40	132 (41.4)	41 (41.8)	
>40	89 (27.9)	27 (27.6)	
Age			0.53
Mean	28.8	29.2	
Median	28.0	29.0	

*p*-Values calculated using Wilcoxon rank sum test for continuous variables; chi-square or Fisher exact (*n* < 5) for categorical. Outcome unknown: Patient did not deliver at Regional One Hospital. Where patient data are missing, the denominators may be less than 320 controls/99 telehealth.

SF, severe features; other, delivery outcome unknown.

Variables of interest were then tested for their univariate association with telehealth utilization using logistic regressions. Those variables found to meet the criterion of *p* < 0.2 in the univariate case were introduced into a multivariable logistic model with a limitation for variable retention.

Also, introduced to this model were relevant clinical variables such as anemia, chronic hypertension, and diabetes. Variables were retained in the model with *p*-value <0.1. Odds Ratios with 95% confidence intervals were also calculated. *p*-Values <0.05 were used for statistical significance. All statistical analyses were performed using SAS v9.4.

## Results

The initial search yielded a total of 419 high-risk patients over the study timeframe. There were 320 patients (76.4%) without telehealth visits and 99 patients (23.6%) with telehealth visits. The mean number of telehealth visits per patient in the telehealth group was 1.28, SD = 0.74. Our study population self-identified as 72.5% Black, 16.9%, White, 8.1% Hispanic, and 2.5% identified as Other. Self-reported race was not a factor in choosing telehealth visits (*p* = 0.81).

Other demographic characteristics are further delineated in Table 1. Patients with private insurance coverage were more likely to have telehealth visits included in their care. In addition, maternal BMI and age were not significantly related to having telehealth visits (*p* = 1.00, 0.53). Mode of delivery and pregnancy outcomes were not impacted by telehealth visits.

Table 2 is a comparison of telehealth patients versus controls. Odds ratios >1 indicate that the condition is more common among the telehealth patients than the controls. Patients with asthma, depression, anxiety, or preeclampsia with severe features were more likely to have telehealth visits. In multivariable logistic modeling, anxiety, maternal obesity, and twin pregnancy were shown to be independent factors associated with having telehealth visits (Table 3).

**Table 2.** Univariate Association with Telehealth Utilization Logistic Regression Models

Covariate	Level	*N*	Telehealth odds ratio (95% CI)	*p*
Anemia			1.1 (0.7–2.6)	0.43
Chronic hypertension			0.8 (0.5–1.4)	0.48
Diabetes			1.1 (0.7–1.8)	0.71
Delivery (ref = vaginal)	Cesarean/vaginal	272	0.7 (0.5–1.2)	0.20
Delivery outcome	Demise	5	0.2 (0.0.3–11.9)	0.16
	Still pregnant	35	0.3 (0.1–0.9)	
	Unknown	64	0.8 (0.4–1.6)	
Diabetes			1.1 (0.7–1.8)	0.71
Insurance (ref = Blue Cross)	Cigna	21	1.7 (0.6–4.6)	0.16
	United health care	161	0.7 (0.4–1.2)	
	Other	89	1.3 (0.7–2.5)	
	Unknown	9	NA	
Asthma			1.8 (1.1–3.2)	0.03
Depression			3.1 (1.4–7.0)	0.01
Anxiety			3.3 (1.4–7.8)	0.01
Twin pregnancy			2.8 (0.9–9.6)	0.09
Preeclampsia with SF			1.0 (0.3–3.0)	0.93
Thyroid disorder			1.9 (0.7–4.8)	0.20
Other comorbidity			0.7 (0.5–1.2)	0.18

CI, confidence interval; NA, not applicable.

**Table 3.** Multivariable Association with Telehealth Utilization Optimized Logistic Regression

Covariate	Telehealth odds ratio (95% CI)	*p*
Asthma	1.7 (0.9–3.0)	0.08
Anxiety	3.3 (1.4–8.1)	0.01
Maternal obesity	2.8 (1.8–4.6)	<0.0001
Twin pregnancy	3.6 (1.0–12.9)	0.04

There were a total of 19 patients who had multiple (>1) telehealth visits. Overall, 37.6% of those patients delivered at term, 10.5% delivered late preterm (>35 weeks), 5.2% delivered preterm (32 weeks), and 10.5% were lost to follow-up. There were no fetal demises noted. Overall, 31.5% delivered vaginally, 42.1% delivered by cesarean section. 10.5% were delivered at an outside facility, 5.2% were not delivered at the time of the study, and 5.2% were lost to follow-up. Pregnancy complications among the patients include: 31.5% chronic hypertension, 15.7% pregestational diabetes, 5.3% gestational diabetes, 26.3% asthma, 15.7% depression, 15.7% anxiety, 42.1% morbid obesity, and 10.5% multifetal pregnancy.

## Discussion

Although the pandemic has increased telehealth utilization, there are still challenges to reach all patient populations. In this study, patients were from a combination rural and urban populations who had care from a tertiary care center. Our study suggested that patients with certain comorbidities in a high-volume academic MFM practice were more likely to have telehealth visits. Visits were initially scheduled by Resident Physicians, Nurse Practitioners, and Providers.

However, patients were subsequently given the option of telehealth versus in-person visits. We analyzed a number of variables that granted us the opportunity to generate a comprehensive review of the benefit of incorporating telehealth visits to aid in the care of high-risk obstetric patients. Factors such as anxiety, asthma, or twin pregnancy may have increased patients' interest in making use of telehealth appointments.

Patients with anxiety may have preferred to avoid being examined in clinic during the pandemic due to viral exposure concerns; however, it is not immediately obvious why patients with asthma, obesity, or a twin pregnancy were more inclined to pursue telehealth visits. Telehealth may meet these patients' needs more readily than in-person visits and they may be prematurely accustomed to telehealth visits by previous mental health sessions, or patients with more complicated conditions such as diabetes and/or hypertension require more in-person visits due to the complexity of the visits including blood pressure or glycemic control logs.

Patients with private insurance such as Blue Cross Blue Shield, Cigna, and United Health Care were more likely to have a telehealth visit than patients with public insurance (Medicaid/Medicare). It is possible that private insurance companies provided better coverage or incentives for telehealth visits. In addition, associated social determinants of health such as barriers to stable internet connection and lack of transportation may have factored into patient decisions to utilize or forgo telehealth appointments. Additional research is needed to further evaluate both incentivization practices of private and public insurance companies and the roles of social determinants of health on telehealth utilization.

The COVID-19 pandemic has impacted life and health care in numerous ways. It forced urgent and creative implementation of telehealth to enable stable continuous care for patients while decreasing further spread of the pandemic where possible. There are limited data on how telehealth has impacted patient care in the field of general obstetrics and gynecology and even less examining high-risk obstetrics patients.

There has been an increase in telehealth offered in obstetrics clinics in many places during the pandemic.^8,9^ One notable study demonstrated that after an initial telehealth appointment, at least half of the patients considered pursuing future telehealth appointments as needed on a regular basis and physicians similarly were interested in offering telehealth options after an initial visit.^10^

The American College of Obstetricians and Gynecologists (ACOG) has made several recommendations regarding telehealth implementation.^9^ While some providers favor the use of t telehealth,^11^ some institutions still face challenges in transitioning and implementing telehealth. Challenges that were found in this transition were barriers with public insurance and additional requirements of the patient (including technological proficiency, language barriers, Wi-Fi and data access, and childcare) in the implementation of telehealth visits.^11^

Fryer et al.^18^ proposed a new model for perinatal health that incorporated telehealth appointments and also discussed ways one health system adapted to telehealth in the time of the pandemic, including having patients purchase blood pressure cuffs, scales, and optionally, fetal dopplers to monitor vital signs from home.^12^ Another study examined how women with maternal obesity utilizing telephone intervention had less gestational weight gain than women in the usual care group.^10^

Telehealth interventions overall improved obstetric outcomes related to smoking cessation and breastfeeding and decreased the need for high-risk obstetric monitoring office visits while maintaining maternal and fetal outcomes.^11,12^

In a literature review of 13 publications conducted by Wu et al.,^19^ virtual care resulted in strong patient and provider satisfaction related to cost savings and convenience, with clinic wait times and cancellation rates also improving. The results support safety equivalence in birth outcomes between in-person prenatal care models and those that integrate virtual visits in both low- and high-risk prenatal populations. Virtual visits provided time and cost savings and reduced appointment cancellations and missed appointments. In addition, virtual visits and postpartum monitoring from home can help health care providers improve the experience for expecting and new parents.^7^

Our study was limited by it being a retrospective study performed at a single academic institution. The reason that some patients chose telehealth over in-person visits is not immediately obvious and we did not question their reasoning for electing for telehealth visits. The findings should be prospectively validated at other institutions. In addition, the small number of patients with multiple telehealth visits may limit the generalizability of the study.

Strengths of this study are that it was completed at a relatively large high-risk practice and number of subjects with a significant proportion of underrepresented minorities and the racial breakdown in population. In summary, our study concludes that there may be certain patients who are more likely to have a combination of in-person and telehealth visits.

The similar delivery outcomes suggest that telehealth is a safe option to combine with in-person visits to maximize scheduling flexibility for patients, physicians, and clinic staff as well as a popular option for those that have utilized telehealth previously. These findings will assist other MFM institutions and are encouraging for the continuation of telehealth visits after the pandemic.

One area that may see improvement is in making scheduled postpartum visits, where ACOG recommends a visit within the first 3 weeks postpartum followed by ongoing care as needed up to 12 weeks, but many patients (40%) are unable to make these appointments, especially in populations with limited resources.^20^

It has been shown by Wu et al.^7,19^ that virtual visits and postpartum monitoring from home can help health care providers improve the experience for expecting and new parents as well as contraception management. Difficulty with lack of follow-up or inappropriate gaps between appointments due to travel, illness, or other difficulties could be ameliorated by implementing a telehealth visit option for patients where clinically appropriate.

## Abbreviations Used

ACOG: American College of Obstetarericians and Gynecologists
BMI: body mass index
CDC: Centers for Disease Control and Prevention
COVID-19: Coronavirus disease 2019
CI: confidence interval
MFM: maternal fetal medicine
SF: severe features

## References

[1] Nesbitt TS, Marcin JP, Daschbach MM, et al. Perceptions of local health care quality in 7 rural communities with telemedicine. J Rural Health 2005;21(1):79–85; doi: 10.1111/j.1748-0361.2005.tb00066.x 15667014

[2] Davis AM, Sampilo M, Gallagher KS, et al. Treating rural pediatric obesity through telemedicine vs. telephone: Outcomes from a cluster randomized controlled trial. J Telemed Telecare 2016;22(2):86–95; doi: 10.1177/1357633X15586642 26026186 PMC4830380

[3] Flodgren G, Rachas A, Farmer AJ, et al. Interactive telemedicine: Effects on professional practice and health care outcomes (review). Cochrane Database Syst Rev 2015;(9); doi: 10.1002/14651858 PMC6473731 26343551

[4] Kahn JM. Virtual visits—Confronting the challenges of telemedicine. N Engl J Med 2015;372(18):1415; doi: 10.1056/NEJMp1502419 25923547

[5] Di Mascio D, Khalil A, Saccone G, et al. Outcome of coronavirus spectrum infections (SARS, MERS, COVID-19) during pregnancy: A systematic review and meta-analysis. Am J Obstet Gynecol MFM 2020;2(2); doi: 10.1016/j.ajogmf.2020.100107. PMC7104131 32292902

[6] Centers for Disease Control and Prevention. COVID-19: Telemedicine—What Does It Mean and Why Should You Care? Centers for Disease Control and Prevention. Available from: https://www.cdc.gov/coronavirus/2019-ncov/global-covid-19/telemedicine.html [Last accessed: April 3, 2022].

[7] Butler Tobah YS, LeBlanc A, Branda ME, et al. Randomized comparison of a reduced-visit prenatal care model enhanced with remote monitoring. Am J Obstet Gynecol 2019;221(6):638.e1–638.e8; doi: 10.1016/j.ajog.2019.06.034 10.1016/j.ajog.2019.06.034 31228414

[8] Abebe SY, Goldsby EA, Renbarger KM. Telehealth for pregnant women with opioid use disorder: A theory-based approach. J Psychosoc Nurs Ment Health Serv 2020;58(12):13–20; doi: 10.3928/02793695-20201112-03. 33238022

[9] Madden N, Emeruwa UN, Friedman AM, et al. Telehealth uptake into prenatal care and provider attitudes during the COVID-19 pandemic in New York City: A quantitative and qualitative analysis. Am J Perinatol 2020;37(10):1005–1014; doi: 10.1055/s-0040-1712939 32516816 PMC7416212

[10] Implementing Telehealth in Practice: ACOG committee opinion summary, number 798. Obstet Gynecol 2020;135(2):493–494; doi: 10.1097/AOG.0000000000003672 31977794

[11] Pflugeisen BM, McCarren C, Poore S, et al. Virtual visits: Managing prenatal care with modern technology. MCN Am J Matern Child Nurs 2016;41(1):24–30; doi: 10.1097/NMC.0000000000000199. 26474477

[12] Pflugeisen BM, Mou J. Patient satisfaction with virtual obstetric care. Matern Child Health J 2017;21(7):1544–1551; doi: 10.1007/s10995-017-2284-1 28176034

[13] Whittington JR, Ramseyer AM, Taylor CB. Telemedicine in low-risk obstetrics. Obstet Gynecol Clin North Am 2020;47(2):241–247; doi: 10.1016/j.ogc.2020.02.006 32451015

[14] Kern-Goldberger AR, Srinivas SK. Telemedicine in obstetrics. Clin Perinatol 2020;47(4):743–757; doi: 10.1016/j.clp.2020.08.007 33153659

[15] Peahl AF, Novara A, Heisler M, et al. Patient preferences for prenatal and postpartum care delivery: A survey of postpartum women. Obstet Gynecol 2020;135(5):1038–1046; doi: 10.1097/AOG.0000000000003731 32282598 PMC7183878

[16] DeNicola N, Grossman D, Marko K, et al. Telehealth interventions to improve obstetric and gynecologic health outcomes: A systematic review. Obstet Gynecol 2020;135(2):371–382; doi: 10.1097/AOG.0000000000003646 31977782 PMC7012339

[17] Limaye MA, Lantigua-Martinez M, Trostle ME, et al. Differential uptake of telehealth for prenatal care in a large New York City academic obstetrical practice during the COVID-19 pandemic. Am J Perinatol 2021;38(3):304–306; doi: 10.1055/s-0040-1721510 33302308

[18] Fryer K, Delgado A, Foti T, et al. Implementation of obstetric telehealth during COVID-19 and beyond. Matern Child Health J 2020;24:1104–1110; doi: 10.1007/s10995-020-02967-7 32564248 PMC7305486

[19] Wu KK, Lopez C, Nichols M. Virtual visits in prenatal care: An integrative review. J Midwifery Womens Health 2022;67(1):39–62; doi: 10.1111/jmwh.13284 34767317

[20] ACOG Committee Opinion No. 736: Optimizing postpartum care. Obstet Gynecol 2018;131(5):e140–e150; doi: 10.1097/AOG.0000000000002633 29683911

